# Production of *N*-acetyl-D-neuraminic acid using two sequential enzymes overexpressed as double-tagged fusion proteins

**DOI:** 10.1186/1472-6750-9-63

**Published:** 2009-07-09

**Authors:** Tzu-Hsien Wang, Ying-Yin Chen, Hsin-Hung Pan, Feng-Pao Wang, Chung-Hsien Cheng, Wen-Chien Lee

**Affiliations:** 1 Department of Chemical Engineering, National Chung Cheng University, Chiayi, 621, Taiwan; 2 Institute of Molecular Biology, National Chung Cheng University, Chiayi, 621, Taiwan

## Abstract

**Background:**

Two sequential enzymes in the production of sialic acids, N-acetyl-D-glucosamine 2-epimerase (GlcNAc 2-epimerase) and *N*-acetyl-D-neuraminic acid aldolase (Neu5Ac aldolase), were overexpressed as double-tagged gene fusions. Both were tagged with glutathione S-transferase (GST) at the N-terminus, but at the C-terminus, one was tagged with five contiguous aspartate residues (5D), and the other with five contiguous arginine residues (5R).

**Results:**

Both fusion proteins were overexpressed in *Escherichia coli *and retained enzymatic activity. The fusions were designed so their surfaces were charged under enzyme reaction conditions, which allowed isolation and immobilization in a single step, through a simple capture with either an anionic or a cationic exchanger (Sepharose Q or Sepharose SP) that electrostatically bound the 5D or 5R tag. The introduction of double tags only marginally altered the affinity of the enzymes for their substrates, and the double-tagged proteins were enzymatically active in both soluble and immobilized forms. Combined use of the fusion proteins led to the production of *N*-acetyl-D-neuraminic acid (Neu5Ac) from *N*-acetyl-D-glucosamine (GlcNAc).

**Conclusion:**

Double-tagged gene fusions were overexpressed to yield two enzymes that perform sequential steps in sialic acid synthesis. The proteins were easily immobilized via ionic tags onto ionic exchange resins and could thus be purified by direct capture from crude protein extracts. The immobilized, double-tagged proteins were effective for one-pot enzymatic production of sialic acid.

## Background

N-acetyl-D-neuraminic acid (Neu5Ac) is the most abundant terminal carbohydrate in glycoconjugates (glycoproteins and glycolipids) and has great commercial value. It is an acidic sugar with a prominent role in numerous biological activities, including anti-viral and anti-bacterial defensive functions. Neu5Ac is the starting material in the manufacture of the anti-influenza virus agent Zanamivir (Relenza) [1]. To enzymatically produce Neu5Ac from N-acetyl-D-glucosamine (GlcNAc), a two-enzyme, sequential system of GlcNAc 2-epimerase (EC 5.1.3.8) and Neu5Ac aldolase (EC 4.1.3.3) is commonly used [2-5]. GlcNAc 2-epimerase catalyzes the inter-conversion of GlcNAc to N-acetyl-D-mannosamine (ManNAc), which then reacts with pyruvate to form Neu5Ac through the enzymatic action of Neu5Ac aldolase [6,7]. GlcNAc 2-epimerase has been found in mammals (humans, rats and pigs) and in the unicellular cyanobacterium *Synechocystis *sp. strain PCC6803. The mammalian GlcNAc 2-epimerase has been defined as a renin-binding protein, with the ability to bind rennin and mask protease activity, similar to cellular rennin inhibitors [8-10]. In addition to the renin-binding version from porcine kidney, GlcNAc 2-epimerase has also been obtained through overexpression from *Synechocystis *sp., and is used in practical applications to catalyze GlcNAc epimerization [11]. The second enzyme in Neu5Ac production, Neu5Ac aldolase, was previously named N-acetylneuraminate lyase and found to be present in *Escherichia coli *K12 and other *E. coli *strains [12-15], as well as a wide variety of other sources, including pathogenic bacteria [16]. The *E. coli *gene for Neu5Ac has been overexpressed for Neu5Ac production from ManNAc and pyruvate [17] and for the production of 2-keto-3-deoxy-D-*glycero*-D-*galacto*-nonopyranulosonic acid (KDN) from D-mannose and pyruvate [18,19].

For large-scale production, enzymes are usually used in immobilized form to allow easy enzyme reuse and product recovery. Enzymes are typically immobilized on solid supports through a covalent linkage. Based on this technology, Neu5Ac aldolase has been immobilized on Eupergit-C for Neu5Ac production [17] and has also been retained in membrane reactors using ultrafiltration membranes [18]. This paper describes the construction of gene fusions for expressing GlcNAc 2-epimerase and Neu5Ac aldolase, both with an N-terminal glutathione S-transferase (GST) tag, and a C-terminal tag of five contiguous aspartate residues (5D) on GlcNAc 2-epimerase and five contiguous arginine residues (5R) on Neu5Ac aldolase. The resulting overexpressed, double-tagged proteins, termed GST-GlcNAc 2-epimerase-5D and GST-Neu5Ac aldolase-5R, were purified and immobilized for enzymatic reaction. The overexpressed proteins in crude extracts could also be directly used for immobilization via the polyionic tags.

## Results

### Expression of double-tagged GlcNAc 2-epimerase

**Figure 1:**
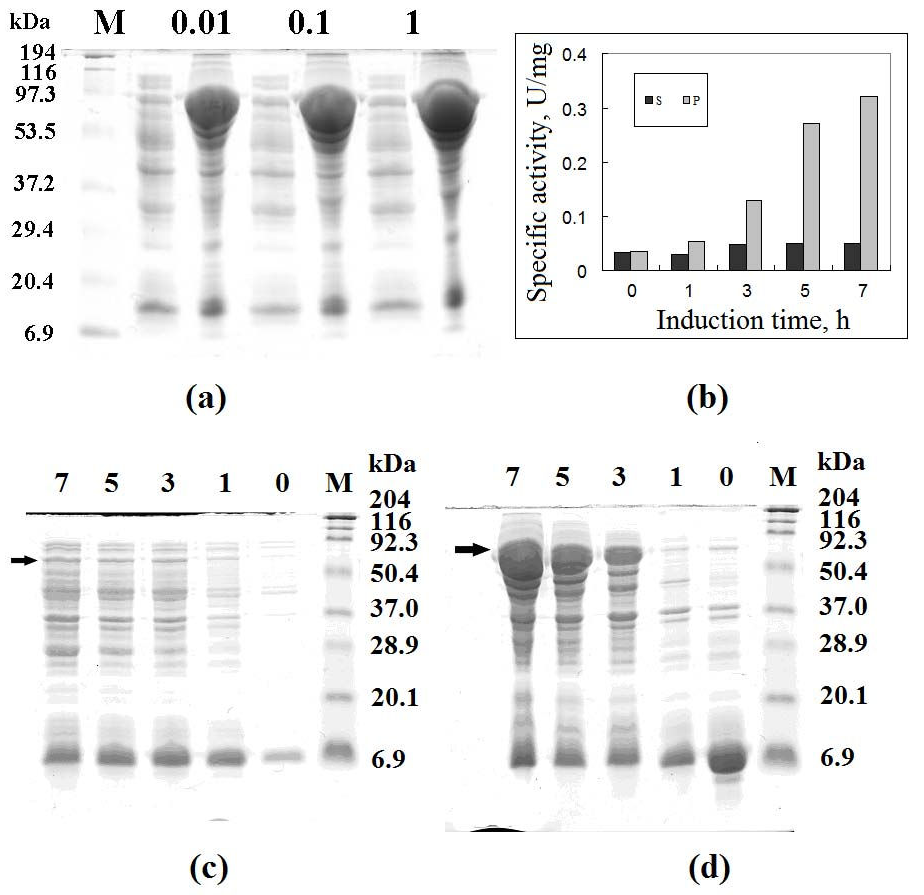
**SDS-PAGE analysis and activity assay of GST-GlcNAc 2-epimerase-5D**. (a) Samples are the supernatant (left lanes) and precipitate (right lanes) of cell extracts from cultures induced with different concentrations of IPTG (0.01, 0.1 and 1 mM) at 28°C for 7 h. (b) Specific activity in the supernatant and precipitate of cell extracts from cultures induced with 0.01 mM IPTG at 28°C for 0 to 7 h. The enzymatic activity of GlcNAc 2-epimerase was based on the formation of GlcNAc from ManNAc. SDS-PAGE analysis of proteins in the supernatant (c) and precipitate (d) of cell extracts from cultures at different induction times is also shown. *M *denotes marker proteins; arrows indicate the positions of fusion proteins.

A 1209 bp PCR fragment encoding the *Synechocystis *sp. PCC6803 GlcNAc 2-epimerase gene, plus a sequence encoding 5D, was cloned into pGEX-2TK between the *Bam*HI and *Eco*RI sites. The resulting plasmid contained a in-frame fusion of GST, GlcNAc 2-epimerase and 5D, and was confirmed by DNA sequencing. Under the inducing conditions indicated in Methods, *E. coli *BL21 harboring the gene fusion produced a significant level of double-tagged GlcNAc 2-epimerase when grown to an OD_600 _of approximately 1.0 and treated with IPTG to induce gene expression. As shown in Figure 1a, the expression level was nearly independent of IPTG concentration between 0.01 to 1 mM, when induction was for 7 h. The fusion protein was largely insoluble (dark diffusive band in lane P for all cases). At every IPTG concentration, only a low level of fusion protein was seen in the supernatant fractions (S) of crude protein extracts.

The expression level of fusion protein increased significantly with induction time, as shown in Figure 1b–1d. One hour after induction, the protein became evident in the S fraction, increased as induction time reached to 3 h, and remained somewhat constant through 5–7 h of induction. In contrast, protein in the P fraction dramatically increased after 3 h, and increased proportionally with time of induction afterward. The specific GlcNAc 2-epimerase activity of protein in the S fraction increased slightly after 3 h of induction, to about 0.05 U/mg when measured as the conversion of ManNAc to GlcNAc, and remained unchanged thereafter. P fractions containing the overexpressed proteins had relatively higher activities. As shown in Figure 1b, the specific activity increased by approximately 2.5 fold in the P fraction after 3 h induction, and after 7 h, the specific activity had risen to 0.32 U/mg, corresponding to approximately 6.5 times the activity of the soluble fractions. These data suggest that the proteins in the precipitate were active, and formed aggregates because their concentrations were much higher than the protein solubility. P fraction samples were prepared by re-dissolving the precipitate of the crude protein extract with 5 ml deionized water. The tremendous increase in specific activity in the P fraction was mainly due to the enrichment of overexpressed double-tagged protein in the precipitated form.

Using the Compute pI/Mw tool in the ExPASy Proteomics Server , the theoretical molecular mass for GST-GlcNAc 2-epimerase-5D was predicted to be 73.2 kDa. The introduction of the GST tag allows the double-tagged fusion protein to be purified from the protein extract using conventional affinity methods. After GSH-affinity purification, the purified GST-GlcNAc 2-epimerase-5D had a specific activity of 11.5 U/mg protein using ManNAc as the substrate. Previous studies on GlcNAc 2-epimerase indicated that this enzyme has a pH optimum of 8 [20], with high activity at pH 7–8, but no activity below pH 6. Since the theoretical pI for native GlcNAc 2-epimerase is 5.59, this means the enzyme is likely to be active only in deprotonized conditions, when pH>pI. The addition of GST to generate GST-GlcNAc 2-epimerase, including the linker sequences, led to a theoretical increase in pI to 5.89. We thus designed a 5D tag at the C-terminus to bring the theoretical pI back to 5.59.

The introduction of tags not only increased the protein solubility, but also altered the thermal stability. A previous study showed that the optimal temperature for GlcNAc 2-epimerase from *Synechocystis *sp. PCC6803 is 37°C [11]. The double-tagged fusion protein was determined to have an optimal temperature of 50°C, suggesting a higher operation temperature was possible for the application of double-tagged GlcNAc 2-epimerase to sialic acid production. The Michaelis constant, *K*_*m*_, for purified GST-GlcNAc 2-epimerase was determined to be 7.7 mM using ManNAc as substrate. This value was slightly higher than the 4.76 mM determined earlier [11] for native GlcNAc 2-epimerase from the same source. Pyruvate is a competitive inhibitor for this enzyme. The inhibition constant of pyruvate for GST-GlcNAc 2-epimerase-5D was 31 mM, which was very close to the 36 mM determined for single-tagged protein GST-GlcNAc 2-epimerase [20].

### Expression of double-tagged Neu5Ac aldolase

Sequences (909 bp) encoding Neu5Ac aldolase and 5R were amplified from *E. coli*. The PCR product was purified and cloned into pGEM-T Easy Vector, before excision and insertion into pGEX-2TK between the *Bam*HI and *Eco*RI sites. This construction created a fusion of Neu5Ac aldolase tagged with GST at the N-terminus and 5R at the C-terminus, with the gene for the double-tagged protein under the regulation of the *tac *promoter. The fusion protein contains a total of 535 amino acid residues comprising Neu5Ac aldolase, the two tags, and the sequence linking GST and Neu5Ac aldolase. Non-tagged Neu5Ac aldolase contains 297 amino acid residues and has a computed MW of 32.6 kDa and theoretical pI value of 5.61, while the computed MW and pI for the fusion protein are 60.4 kDa and 6.84.

**Figure 2:**
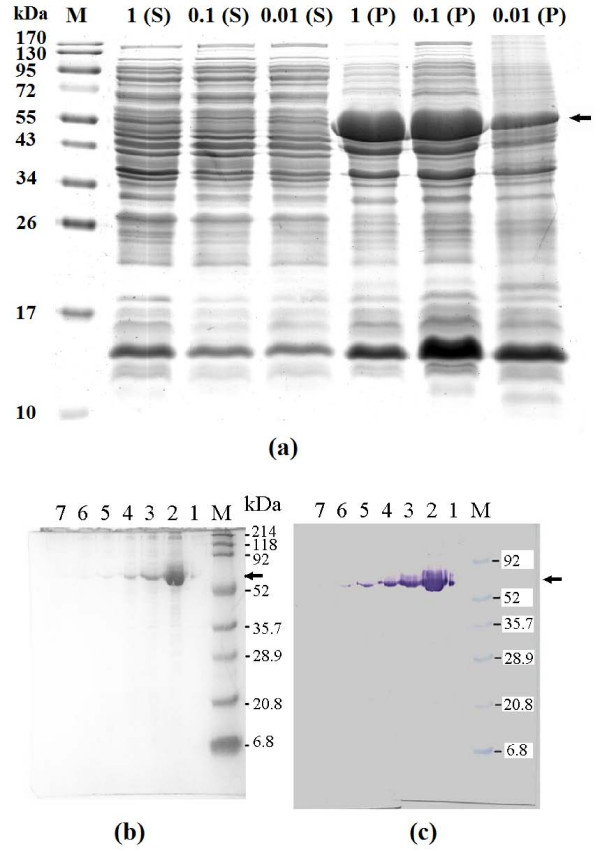
**SDS-PAGE analysis and Western blotting of GST-Neu5Ac aldolase-5R**. For panel (a), samples are the supernatant (S) and precipitate (P) of cell extracts from cultures induced with 0.01, 0.1 or 1 mM IPTG at 28°C for 7 h. In panel (b) and (c), samples are fractions collected from a GSH-affinity column. Lane M, molecular size makers; lane 1–7, fractions 1–7. Arrows indicate the purified fusion proteins.

Similar to GST-GlcNAc 2-epimerase-5D, GST-Neu5Ac aldolase-5R could also be overexpressed in *E. coli*. SDS-PAGE results for GST-Neu5Ac aldolase-5R (Figure 2a) were similar to those for GST-GlcNAc 2-epimerase-5D. Unlike the induction of GST-GlcNAc 2-epimerase-5D, however, the expression level of GST-Neu5Ac aldolase-5R depended on inducer concentration. The use of 0.01 M IPTG led to a significantly lower level of protein expression than 0.1 and 1 mM. The fusion protein was largely insoluble (dark diffusive band in lane P). The band showing the level of soluble protein (lane S) was indistinct, suggesting a relatively low solubility for GST-Neu5Ac aldolase-5R.

Overexpression of GST-Neu5Ac aldolase-5R was confirmed by analyzing the purified fusion protein after affinity purification. Figure 2b and 2c show the SDS-PAGE analysis and Western blotting of GST-Neu5Ac aldolase-5R from GSH-affinity column fractions. Western blotting using antibody against the GST tag showed that the molecular mass of double-tagged protein was close to the theoretical value of 60.4 kDa.

In the fused construct in plasmid pGEX-2TK, a thrombin recognition site lies between GST and Neu5Ac aldolase, so the GST tag could be released from the fusion protein by thrombin digestion. Cleavage of the double-tagged fusion protein by thrombin resulted in two proteins, GST and Neu5Ac aldolase-5R. Both the double-tagged fusion protein GST-Neu5Ac aldolase-5R, and the GST-released fusion protein Neu5Ac aldolase-5R were able to bind SP Sepharose. The typical adsorption curve displayed a change in adsorbed fusion protein with time. A sharp decrease of proteins in solution was observed within the first few hours, followed by a very slow decrease during the immobilization process. This was seen using two preparations of the purified fusion proteins. Although the theoretical pI values for GST-Neu5Ac aldolase-5R and Neu5Ac aldolase-5R with their linker sequences are 6.84 and 8.27, the experimental pI values were estimated as 8.3 and 8.8 from two-dimensional gel electrophoresis. Thus, they could easily be immobilized on the cationic exchanger SP Sepharose.

### Immobilization of fused proteins

**Figure 3:**
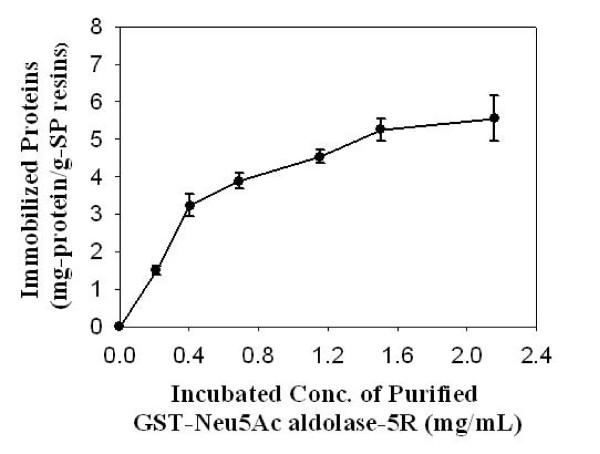
**Immobilization of purified double-tagged GST-Neu5Ac aldolase-5R using cationic exchanger SP Sepharose**. The amount of fusion protein immobilized varies with the amount of fusion protein used. Prior to immobilization, GST-Neu5Ac aldolase-5R was purified using a GSH-affinity column. Data are an average of four independent experiments.

For the production of sialic acid, Neu5Ac, using GlcNAc 2-epimerase and Neu5Ac aldolase in a single pot, both enzymes should be active at the operational conditions, including pH and temperature. A pH in the range of 7–7.5 should be optimal, because in that range, the double-tagged Neu5Ac aldolase would be positively charged, since its pI value was increased by the introduced 5R tag. This allowed the fusion protein to be easily adsorbed onto cationic SP Sepharose resins. In contrast, the double-tagged GlcNAc 2-epimerase would be negatively charged and able to bind to the anionic exchanger Q Sepharose. Immobilization of the GST affinity-purified fusion proteins onto ion exchange resins was achieved in a few hours. Greater concentrations of proteins mixed with the ion exchanger caused a longer adsorption time before adsorption equilibrium was reached. If a ten-hour adsorption was used, the amount of fusion protein in the incubated solution could be increased, leading to more adsorbed protein on the resin (Figure 3). When the applied concentration was 0.2 mg/ml, about 90% of the purified fusion protein could be immobilized. When the applied concentration was 2.2 mg/ml, the immobilized protein approached 5.6 mg protein/g resin, corresponding to immobilization of approximately 30% of the proteins. Immobilization led to a reduction in enzymatic activity of about 25%, based on the specific activity of purified protein as 100%.

Time courses of immobilization of double-tagged GlcNAc 2-epimerase on Q Sepharose were similar to those for immobilization of double-tagged Neu5Ac aldolase on SP Sepharose. A similar pattern (Figure 3) was seen for the influence of the applied concentration of purified GST-GlcNAc 2-epimerase-5D on the amount of protein bound to Q Sepharose.

**Figure 4:**
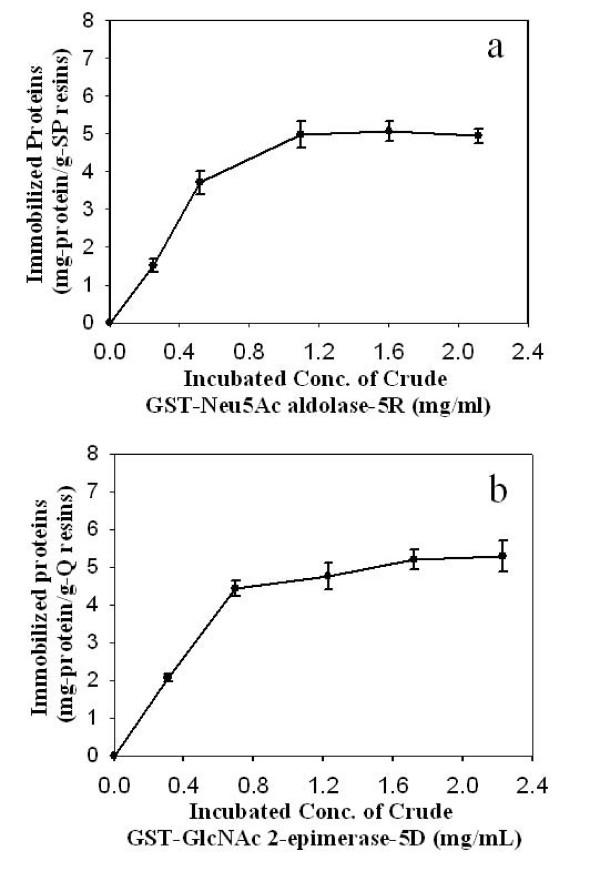
**Influence of initial protein concentration on the amount of immobilized protein**. Results are from a 10-h, direct-capture adsorption of GST-Neu5Ac aldolase-5R (a) or GST-Neu5Ac aldolase-5R (b), from crude protein extracts. In each experiment, 10 ml of protein solution was incubated with 1.2–1.3 g (wet weight) of ion-exchange resin for 10 h.

Since the lysis of cells overexpressing the tagged protein resulted in a large amount of enzymatically active protein in the precipitate, repeated lysis (typically three times) was employed to increase the volume of lysis buffer as well as the extent of cell disruption and protein dissolution. All supernatants were pooled and used as the crude protein extract for fusion protein immobilization. By using the ionic tags, direct capture of fusion protein from the crude protein extract was very effective. As shown in Figure 4, the amount of protein immobilized on the resins increased with the loaded concentration of protein. For double-tagged Neu5Ac aldolase binding to SP Sepharose, the amount of bound protein approached 5.0 mg protein/g-resin when the crude extract protein concentration was 1.2 mg/ml. The bound protein concentration remained at this level even if the applied concentration was higher, suggesting saturation of protein adsorption. Like the adsorption of purified double-tagged Neu5Ac aldolase to the same ion exchange resin, the fraction of crude protein adsorption decreased with increased protein loading concentration. If the loaded concentration of protein was 2.1 mg/ml, only 30% of proteins in the crude extract were immobilized on the resin. A purification effect was seen for selective immobilization of fusion proteins through their ionic interactions. When a crude, 1.1 mg/ml double-tagged Neu5Ac aldolase solution with a specific activity of 0.75 U/mg was applied to SP Sepharose, the protein eluted from the ion exchanger with 1 N NaCl possessed a specific activity of 2.5 U/mg.

Similar adsorption behavior was observed for the immobilization of GST-GlcNAc 2-epimerase-5D on Q Sepharose. When the loading concentration of crude protein was 2.2 mg/ml, the saturated adsorbed density of protein was 5.3 mg protein/g resin. Under these conditions, about 30% of proteins from the crude extract were immobilized on the resin. Since the protein was not purified prior to immobilization, the specific activity of the immobilized fusion protein was lower than that of immobilized, purified fusion protein. Based on the activity of immobilized, purified GST-Neu5Ac aldolase-5R, the specific activity of immobilized GST-Neu5Ac aldolase-5R via direct capture from crude extract was only 21%. The specific activity of immobilized GST-GlcNAc 2-epimerase-5D via direct capture from crude extract was 37% of the activity of immobilized, purified GST-GlcNAc 2-epimerase-5D. However, the specific activity of immobilized protein obtained by direct capture from crude extract was still much higher than that of protein from crude extract, suggesting a purification effect from selective immobilization through the ionic interaction. When a 1.1. mg/ml crude GST-GlcNAc 2-epimerase-5D solution with a specific activity of 1.6 U/mg, using GlcNAc as the substrate, was applied to Q Sepharose, protein from the ion exchanger with 1 N NaCl possessed a specific activity of 3.4 U/mg.

### Production of Neu5Ac using two immobilized double-tagged fusion proteins

Both the soluble and immobilized forms of double-tagged 2-epimerase-5D were effective for the epimerization of GlcNAc and ManNAc. Using GlcNAc as the substrate, the conversion rate catalyzed by purified GST-GlcNAc 2-epimerase-5D increased with the dose of fusion protein and the reaction time. The double-tagged Neu5Ac aldolase immobilized on the cationic exchanger SP Sepharose was able to catalyze the formation of KDN from D-mannose and pyruvate [21].

**Figure 5:**
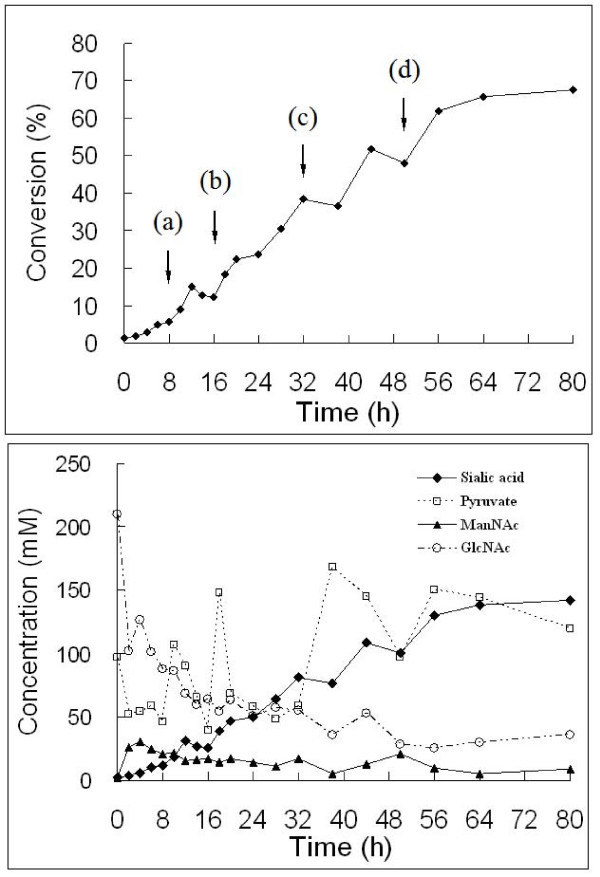
**Production of Neu5Ac from GlcNAc using immobilized GST-GlcNAc 2-epimerase-5D and GST-Neu5Ac aldolase-5R**. Conversion (upper panel) and time-course (lower panel) of GlcNAc, ManNAc, pyruvate, and Neu5Ac (sialic acid) concentrations in the synthesis of Neu5Ac from GlcNAc and pyruvate. Pyruvate was added at (a) 8 h (0.6 ml, 5 M), (b) 16 h (1.2 ml, 5 M), (c) 32 h (1.2 ml, 5 M), and (d) 50 h (0.6 ml, 5 M), and the temperature shifted from 30°C to 20°C at 32 h. Other reaction conditions are indicated in Methods.

Figure 5 shows a typical, small-scale run of Neu5Ac production using GST-GlcNAc 2-epimerase-5D immobilized on Q Sepharose (3 g, corresponding to 2430 U/l) and GST-Neu5Ac aldolase-5R immobilized on SP Sepharose (15 g, corresponding to 7161 U/l) as the biocatalysts. Since pyruvate is a substrate for the second reaction but an inhibitor for enzyme of the first reaction, its concentration must be kept below 100 mM, although not too low, during the reaction. Pyruvate was thus added to the reaction whenever its concentration went below 50 mM. Furthermore, a low temperature was chosen to favor equilibrium to Neu5Ac, although this resulted in a slower reaction rate. Shifting the temperature from 30 to 20°C in the middle of the coupling reactions might lead to the gain of high conversion yield, as suggested in the literature [4]. Finally, a conversion of about 68% based on the production of Neu5Ac from GlcNAc on a molecular basis was achieved in 80 h, corresponding to a volumetric productivity of 0.52 g Neu5Ac l^-1 ^h^-1^. If the reaction was stopped at 56 h, conversion was 62%, corresponding to a productivity of 0.69 g Neu5Ac l^-1 ^h^-1^. An experimental run using 2000 U/l GlcNAc 2-epimerase and 8000 U/l Neu5Ac aldolase on concentrated substrates 816 mM GlcNAc and 483 mM pyruvate at 30°C, resulted in a conversion of 68% at 140 h, corresponding to a productivity of 0.92 g Neu5Ac l^-1 ^h^-1^. A final conversion of 77% was seen after 240 h of reaction, corresponding to a productivity of 0.60 g Neu5Ac l^-1 ^h^-1^) [3]. Our results suggest that the coupled enzymatic system using immobilized double-tagged proteins has practical potential. In the present study, however, the ratio of pyruvate to GlcNAc in the late phase of reaction was kept at a higher level than described previously [3]. Further experiments need to be carried out to avoid using a large excess of pyruvate.

In another Neu5Ac synthesis reaction, combined doses of 1850 U/l GST-GlcNAc 2-epimerase-5D immobilized on Q Sepharose and 1682 U/l GST-Neu5Ac aldolase-5R immobilized on SP Sepharose were used as biocatalysts. A conversion of 35% could be achieved in 28 h, corresponding to a productivity of 0.77 g Neu5Ac l^-1 ^h^-1 ^at 30°C, on the same initial concentration of substrates.

## Discussion

Tagged fusion proteins are frequently used for the purification of overexpressed proteins. In addition to enhancing purification through the use of tags, or fusion partners, that can bind specifically to affinity resins, the introduction of a tag can also improve the solubility of the target protein and facilitate its detection during expression and purification. The addition of a fusion partner can result in high-level expression of the target protein. In this work, we found that introduced tags could not only provide a site for specific purification and immobilization, but also offered the possibility of adjusting the physical properties, such as pI value, of the target protein. GlcNAc 2-epimerase showed a narrow pH range of enzymatic activity. The introduction of GST and 5D tags at N- and C-termini maintained the theoretical pI of the double-tagged GlcNAc 2-epimerase at the same value as the native protein. The fusion form of the enzyme was active at reaction conditions of pH 7–7.5 that deprotonized the enzyme protein. The introduced GST and 5R tags increased the theoretical pI of native Neu5Ac aldolase from 5.61 to 6.84. The two enzymes, overexpressed as double-tagged fusion proteins, could easily be isolated from cell extracts, and possessed enzymatic activity at operational conditions.

The introduction of tags did not alter significantly the kinetic properties of GlcNAc 2-epimerase and Neu5Ac aldolase. For GlcNAc 2-epimerase, the GST and 5D tags increased the Michaelis constant from 4.76 mM for the native protein to 7.7 mM for fusion protein, when ManNAc was used as the substrate. Also, the introduced tags did not change the inhibition constant of pyruvate for GlcNAc 2-epimerase. Similarly, the introduction of GST and 5R tags only marginally altered the kinetic properties of Neu5Ac aldolase. The Michaelis constant of purified GST-Neu5Ac aldolase-5R was 6.9 mM using Neu5Ac as the substrate, which was slightly higher than the 2.5–3.6 mM reported in the literature [22,23] for native *E. coli *Neu5Ac aldolase. When only the GST tag was introduced into Neu5Ac aldolase, the *K*_*m *_value for the single-tagged protein was 2.8 mM against Neu5Ac [19]. The second, highly charged 5R tag only slightly altered the affinity of the enzyme for its substrate. These findings suggest that the active sites of fused proteins were not hindered by tags at both the N- and C-termini. The strategy proposed in this work could not be generalized and adapted for other enzymatic systems in which the enzyme proteins do not have well-exposed N- and C-termini, or the introduction of tags severely changes the conformation and activity of the protein.

Both fusion proteins were overexpressed via pGEX-2TK, with the fusion protein genes under control of the *tac *promoter. Induction time had a greater influence on fusion protein expression level than IPTG concentration. A low concentration of 0.01 mM inducer seemed sufficient for the induction of GST-GlcNAc 2-epimerase-5D overexpression. For overexpression of GST-Neu5Ac aldolase-5R, however, the inducer concentration influenced the expression level and fusion protein was expressed at a very low level when no inducer was present. This leak expression is not uncommon in the pGEX expression system. The overexpressed proteins in the precipitate had a relatively high activity, suggesting that most overexpressed proteins were active but formed aggregates, with the concentration of proteins exceeding their solubility.

Although the GST tag of 220 aa, with a theoretical pI of 5.91 and MW of 25,700 Da, has been widely used for protein purification and immobilization, we used it here for pI adjustment and increasing protein solubility. Direct capture of fusion proteins from crude protein extracts could easily be achieved using the ionic tags. The immobilized preparation from direct binding of overexpressed fusion protein was thus useful for practical applications since it possessed high enzymatic activity and allowed the purification step to be eliminated. The adsorption behaviors for both fusion proteins were very similar. For double-tagged Neu5Ac aldolase on SP Sepharose, the amount of bound protein approached 5.0 mg protein/g resin, while the maximum binding capacity of GST-GlcNAc 2-epimerase-5D on Q Sepharose was 5.3 mg protein/g-resin. For both double-tagged proteins, a purification effect after selective immobilization through ionic interaction with the ion exchange resins was observed.

In this work, two double-tagged proteins were immobilized onto ion exchangers for practical application in Neu5Ac production. The production of Neu5Ac from GlcNAc involves two sequential enzymes, and both could be used in the immobilized state. In a preliminary run, a 68% conversion could be achieved in 80 h using immobilized, double-tagged GlcNAc 2-epimerase and Neu5Ac aldolase, both prepared by direct capturing from crude protein extracts. In this particular enzyme reaction system, many parameters can be adjusted, so the optimal strategy for Neu5Ac production will be subjected to study using mathematical modelling and validated with experimental data [24]. Adjustable parameters include the doses and ratio of the two enzymes, the initial concentration ratio of pyruvate to GlcNAc, the timing of pyruvate addition and temperature programming, among others.

## Conclusion

In summary, for one-pot enzymatic production of sialic acid, we overexpressed two sequential enzymes as double-tagged fusion proteins that were easily immobilized onto ionic exchange resins by direct capture from crude protein extracts. The gene fusions were constructed so that enzyme activities were not altered, and expressed fusion proteins were charged at reaction pH. Experimental results showed that both double-tagged fusions, GlcNAc 2-epimerase and Neu5Ac aldolase, functioned well, and sialic acid could be produced using the two proteins immobilized on anoinic or cationic exchange resins.

## Methods

### Materials

Ampicillin, isopropyl-D-thiogalactopyranoside (IPTG), phenylmethylsufonylfluoride (PMSF) and lysozyme were from MDBio, Inc (Taipei, Taiwan). Neu5Ac (96%) was from TCI (Tokyo, Japan). Triton X-100, N-acyl-D-hexosamine oxidase (AHOX), horseradish peroxidase, adenosine 5'-triphosphate (ATP), and N-acetyl-D-glucosamine (GlcNAc) were from Sigma (St. Louis, MI, USA). 4-Aminoantipyrine was from R.D.H (Seelze, Germany). N-Acetyl-D-mannosamine (ManNAc) and 2,4,6-triiodo-3-hydroxybenzoic acid (HTIB) were from Lancaster (Windham, NH, USA). Reduced glutathione (GSH), pyruvic acid, sodium salt (99+%) and D-mannose (99+%) were from Acros (Geel, Belgium). Glutathione-Uniflow Resin was from Clontech, BD (Palo Alto, CA, USA).

### Construction of the GlcNAc 2-epimerase gene fusion

Isolation of chromosomal DNA from *Synechocystis *sp. PCC6803 (from the Pasteur Institute, Paris, France) was performed as described [25], with modifications. *Synechocystis *sp. grown in 200 ml of medium BG-11 at 25°C with a sun trap were harvested by centrifugation and resuspended in 1 ml deionized water. The cell suspension was frozen in liquid nitrogen and ground into powder which was mixed with 15 ml of extraction buffer consisting of 100 mM Tris-HCl (pH 8.0), 50 mM Na_2_EDTA (pH 8.0), 500 mM NaCl, and 10 mM β-mercaptoethanol. After addition of 1 ml of 20% SDS, the mixture was incubated in a water bath at 65°C for 10 min, before adding 5 ml of 5 M potassium acetate. The supernatant was collected by centrifugation at 26,895 × *g *and mixed with 10 ml 2-propanol before freezing at -20°C for 12 hours. After centrifugation at 20, 201 × *g *for 30 min, the pellet was dried at 30–40°C and dissolved in 0.5 ml deionized water before adding 1 ml of 95% ice-cold ethanol and centrifuging at 23,428 × *g *for 10 min. Finally, the pellet was dried at 40°C and dissolved in 0.5 ml deionized water to yield the chromosomal DNA preparation that was used as PCR template [20].

The GlcNAc 2-epimerase gene was amplified by PCR using DyNAzyme II DNA polymerase (Finnzymes, Espoo, Finland) with primer 1 GATGGATCCATGATTGCCCATCGCCGTCAG and primer 2 GCGGAATTCTTA**ATCATCATCATCATC**ACTAACCGGAAGTTGGAG, where underlined sequences indicate restriction endonuclease sites, and the bolded 15 bases encode the five C-terminal glutamates. The PCR fragment was purified by QIAquick™ PCR purification kit (Qiagen GmbH) and inserted into plasmid vector pGEX-2TK (Amersham Bioscience). Prior to ligation with T4 DNA ligase (New England Biolabs), the PCR fragment and purified plasmid were separately digested with *Bam*HI and *Eco*RI (New England Biolabs). The recombinant plasmid was called pGEX-2TK-2ep-5D and transformed into *E. coli *DH5α. For overexpression, the recombinant plasmid was purified from *E. coli *DH5α using the Min-M™ Plasmid DNA Extraction System kit (Viogene) and retransformed into *E. coli *BL21 (Amersham Bioscience).

### Construction of the Neu5Ac aldolase gene fusion

*E. coli *K12 was cultured in LB media as the source of the N-acetyl-D-neuraminic acid aldolase gene, denoted *nanA*. Primers for PCR amplification of the *nanA *fragment were primer 1 (30-mer): 5'-GAGGGATCCATGGCAACGAATTTACGTGGC and primer 2 (54-mer): TATTATGAATTCTTA**ACGACGACGACGACG**CCCGCGCTCTTGCATCAACTGCTG, where underlined sequences are restriction endonuclease sites, and the 15 bolded bases encodes the five C-terminal arginines. PCR using *HiFi *DNA polymerase (Yeastern Biotech Co., Taipei, Taiwan) was carried out in a GeneAmp™ PCR System 2400 (Perkin Elmer) with denaturation at 94°C for 1.5 min, annealing at 56°C for 3 min and polymerization at 72°C for 2 min. Preheating was at 94°C for 5 min, and extension was at 72°C for 10 min after cycle 30. The PCR product was ligated to pGEM-T Easy Vector (Promega) and the resulting plasmid transformed into *E. coli *DH5α. Plasmid purified from *E. coli *DH5α was digested with *Bam*HI and *Eco*RI to isolate a fragment that was ligated to pGEX-2TK, previously digested with *Bam*HI and *Eco*RI. The resulting plasmid was denoted pGEX-2TK-nanA-5R and transformed into *E. coli *DH5α before transfer to *E. coli *BL 21 for overexpression of double-tagged Neu5Ac aldolase.

### Expression and GST-purification of fusion proteins

Recombinant bacteria were cultured in LB medium (100–200 ml) at 28°C. IPTG (0.01–1 mM) was added at log phase to induce the overexpression of the fusion proteins over one to several hours at 28°C. To characterize fusion protein overexpression, harvested cell pellets were dispersed in 10 ml of PBS buffer, consisting of 140 mM NaCl, 2.7 mM KCl, 10 mM Na_2_HPO_4 _and 1.8 mM KH_2_PO_4 _(pH 7.5) per 100 ml of cell culture [19,20]. Lysozyme and PMSF were added to final concentrations of 1 mg/ml and 2 mM, respectively. The resultant mixture was sonicated at 4°C for 90 min to disrupt cells. A 1% (v/v) Triton X-100 solution in deionized water was added for 60 min at 4°C. Centrifugation at 3000 × *g *for 30 min at 4°C resulted in supernatant protein extract (denoted as S) and a precipitate. For protein and activity assays, the precipitate was dissolved in 5 ml deionized water to generate a sample denoted as P. Fixed volumes of S and P protein solutions were subjected to activity assay and 15% SDS-PAGE analysis. Protein concentration was determined by the Bradford method (Bio-Rad) at 595 nm using bovine serum albumin as the standard.

For the purification and immobilization of overexpressed fusion proteins, crude protein extract was obtained by lysing harvested recombinant *E. coli *cells in lysis buffer consisting of 100 mM NaCl, 50 mM Na_2_HPO_4_, 0.1 mM EDTA, 10 mM β-mercaptoethanol, 0.2% Triton X-100, 25 μg/L PMSF and 40 μg/L lysozyme at a 1:10 volume ratio of lysis buffer to bacterial culture. Lysis was by sonication at 4°C for 30 min and centrifugation at 11,953 × g for 20 min. The precipitate fraction was subjected to lysis again by the same procedure. Supernatants from repeated lysis were combined for the crude protein extract. A 2 ml glutathione resin column (Clontech, BD) was used to purify the double-tagged fusion proteins. An aliquot of clarified crude protein extract were loaded on the column for adsorption of tagged fusion proteins by GST-glutathione affinity. After washing with pre-chilled extraction buffer containing 140 mM NaCl, 10 mM Na_2_HPO_4_, and 1.8 mM KH_2_PO_4 _(pH 7.5), the bound tagged protein was eluted with 10 mM soluble glutathione dissolved in 50 mM Tris-HCl (pH 8.0), collecting 1 ml fractions for analysis by 15% SDS-PAGE and Western blotting.

### Immobilization of fusion proteins

Q Sepharose and SP Sepharose (Amersham Biosciences) were used to immobilize purified double-tagged GlcNAc 2-epimerase (GST-GlcNAc 2-epimerase-5D) and Neu5Ac aldolase (GST-Neu5Ac aldolase-5R), respectively. GSH-purified double-tagged proteins at different concentrations were incubated with the ion exchange resins in test tubes for immobilization, at 10 ml protein solution and approximately 1.2 g ion exchange resin, which corresponded to 3 mL. The test tubes were slowly rotated to allow the mixing between protein and ion exchanger.

Double-tagged proteins were also directly immobilized onto the ion exchange resins described above, via electrostatic interaction between the polyionic tags at the C termini. Ion exchange resins, centrifuged at 4°C to remove free water, were mixed with crude protein extracts adjusted to specified concentrations, for a volume ratio of crude extract to ion exchange resin of 5:1.

### Production of Neu5Ac

For one-step Neu5Ac synthesis, a combination of 2430 U/l of immobilized GST-GlcNAc 2-epimerase-5D and 7161 U/l of immobilized GST-Neu5Ac aldolase-5R was used. The activity of immobilized GST-GlcNAc 2-epimerase-5D was determined using GlcNAc as the substrate. Both immobilized fusion proteins were prepared by direct capture from crude protein extract. Reaction mixtures of 50 ml consisted of GlcNAc 200 mM, pyruvate 100 mM, ATP 10 mM, MgCl_2 _10 mM (pH 7). Pyruvate was added four times over course of the reaction, at 8 h (0.6 ml, 5 M), 16 h (1.2 ml, 5 M), 32 h (1.2 ml, 5 M) and 50 h (0.6 ml, 5 M), and the temperature was shifted from 30°C to 20°C at 32 h. Samples (20 μl) were assayed for GlcNAc, pyruvate, ManNAc, and Neu5Ac in the reaction mixture by HPLC equipped with a RI detector and a Aminex-87H (Bio-Rad) column at mobile phase 5 mM H_2_SO_4_; flow-rate, 0.6 ml/min; temperature, 65°C.

### Activity assay

GlcNAc 2-epimerase activity in the fusion protein was determined based on either the estimation of the GlcNAc formation rate using ManNAc as the substrate, or the formation of ManNAc using GlcNAc as the substrate. For the first method, a colorimetric method developed by Takahashi et al. [26] was employed. The reaction mixture (0.1 ml) containing 0.1 M Tris-HCl (pH 7.5), 40 mM ManNAc, 10 mM MgCl_2_, 4 mM ATP, and 20 μl of enzyme (tagged protein) was incubated at 37°C for 30 min and boiled for 5 min to terminate the reaction. To quantify produced GlcNAc, the sample (20 μl) was mixed with 0.25 ml of solution I (1 mM 4-aminoantipyrin, 0.1% NaN_3_, 0.5 U/ml of AHOX, 5 U/ml of horseradish peroxidase in 0.1 M of pH 7.2 sodium phosphate buffer) and 0.25 ml of solution II (2 mM HTIB and 0.1% NaN_3 _in 0.1 M of pH 7.2 sodium phosphate buffer). After incubation at 37°C for 20 min, absorbance at 515 nm was measured. One unit of activity (U) was defined as the amount of enzyme that catalyzes the formation of one micromole GlcNAc from ManNAc per minute.

In the second approach, GST-GlcNAc 2-epimerase-5D activity was assayed by incubating a protein preparation with 1 ml of assay solution containing 100 mM GlcNAc, 10 mM MgCl_2_, 5 mM ATP in 100 mM Tris-HCl buffer (pH 7.5) for 10 min at 37°C. The reaction was stopped at 100°C for 5 min and the amount of ManNAc produced was determined by HPLC as above. One unit of activity (U) was defined as the amount of enzyme that catalyzes the formation of one micromole ManNAc from GlcNAc per minute.

Neu5Ac aldolase activity of the fusion protein was determined using Neu5Ac as the substrate, measuring the decrease of Neu5Ac. The protein preparation was incubated with 1 ml of Tris-HCl buffer (100 mM, pH 7.5) containing 20 mM Neu5Ac for 10 min at 37°C. The reaction was stopped at 100°C for 5 min and the amount of Neu5Ac consumed determined by HPLC as above. One unit of activity (U) was defined as the amount of enzyme required to cleave 1 μmol of Neu5Ac per minute at 37°C.

## Authors' contributions

T-HW carried out the cloning and expression of GST-GlcNAc 2-epimerase-5D. Y-YC did the cloning and expression of GST-Neu5Ac aldolase-5R. H-HP performed the immobilization of the fusion proteins and production of sialic acid. F-P W and C-HC conducted the experiments on the purification of the fusion proteins. W-CL was in charge of organizing the team, conducting the study, and writing the manuscript.

## References

[B1] Von Itzstein M, Wu W-Y, Kok GB, Pegg MS, Dyason JC, Jin B, Van Phan T, Smythe ML, White HF, Oliver SW, Colman PM, Varghese JN, Ryan DM, Woods JM, Bethell RC, Hotham VJ, Cameron JM, Penn CR (1993). Rational design of potent sialidase-based inhibitors of influenza virus replication. Nature.

[B2] Kragl U, Gygax D, Ghisalba O, Wandrey C (1991). Enzymatic two-step synthesis of N-acetylneuraminic acid in the enzyme membrane reactor. Angew Chem Int Ed Engl.

[B3] Maru I, Ohnishi J, Ohta Y, Tsukada Y (1998). Simple and large-scale production of *N*-acetylneuraminic acid from *N*-acetyl-*D*-glucosamine and pyruvate using *N*-acyl-*D*-glucosamine 2-epimerase and N-acetylneuraminate lyase. Carbohydr Res.

[B4] Lee JO, Yi JK, Lee SG, Takahashi S, Kim BG (2004). Production of N-acetylneuraminic acid from *N*-acetylglucosamine and pyruvate using recombinant human rennin binding protein and sialic acid aldolase in one pot. Enzyme Microb Technol.

[B5] Lee YC, Chien HC, Hsu WH (2007). Production of N-acetyl-D-neuraminic acid by recombinant whole cells expressing *Anabaena *sp. CH1 N-acetyl-D-glucosamine 2-epimerase and *Escherichia coli *N-acetyl-D-neuraminic acid lyase. J Biotechnol.

[B6] Auge C, David S, Gautheron C (1984). Synthesis with immobilized enzyme of the most important sialic acid. Tetrahedron Lett.

[B7] Kim MJ, Henne WJ, Sweers HM, Wong CH (1988). Enzymes in carbohydrate synthesis: *N*-acetylneuraminic acid aldolase catalyzed reactions in preparation of *N*-acetyl-2-deoxy-D-neuraminic acid derivatives. J Am Chem Soc.

[B8] Maru I, Ohta Y, Murata K, Tsukada Y (1996). Molecular cloning and identification of *N*-acyl-*D*-glucosamine 2-epimerase from porcine kidney as a renin-binding protein. J Biol Chem.

[B9] Takahashi S, Takahashi K, Kaneko T, Ogasawara H, Shindo S, Kobayashi M (1999). Human renin-binding protein is the enzyme *N*-acetyl-*D*-glucosamine 2-epimerase. J Biochem.

[B10] Takahashi S, Hori K, Takahashi K, Ogasawara H, Tomatsu M, Saito K (2001). Effects of nucleotides on *N*-acetyl-*D*-glucosamine 2-epimerases (renin-binding proteins): comparative biochemical studies. J Biochem.

[B11] Tabata K, Koizumi S, Endo T, Ozaki A (2002). Production of *N*-acetyl-*D*-neuraminic acid by coupling bacteria expression *N*-acetyl-*D*-glucosamine 2-epimerase and *N*-acetyl-*D*-neuraminic acid synthetase. Enzyme Microb Technol.

[B12] Ohta Y, Watanabe K, Kimura A (1985). Complete Sequence of *E. coli N*-acetylneuraminate lyase. Nucleic Acids Res.

[B13] Aisaka K, Uwajima T (1986). Cloning and constitutive expression of the *N*-acetylneuraminate lyase gene of *Escherichia coli*. Appl Environ Microbiol.

[B14] Rodriguez-Aparicio LB, Ferrero MA, Reglero A (1995). *N*-acetyl-*D*-neuraminic acid synthesis in *Escherichia coli *K1 occurs through condensation of *N*-acetyl-*D*-mannosamine and pyruvate. Biochem J.

[B15] Blayer S, Woodley JM, Lilly MD, Dawson MJ (1996). Characterization of the chemoenzymatic synthesis of NacetylDneuraminic acid (Neu5Ac). Biotechnol Prog.

[B16] Li Y, Yu H, Cao H, Lau K, Muthana S, Tiwari VK, Son B, Chen X (2008). *Pasteurella multocida *sialic acid aldolase: a promising biocatalyst. Appl Microbiol Biotechnol.

[B17] Mahmoudian M, Noble D, Drake CS, Middleton RF, Montgomery DS, Piercey JE, Ramlakhan D, Todd M, Dawson MJ (1997). An efficient process for production of *N*-acetylneuraminic acid using *N*-acetylneuraminic acid aldolase. Enzyme Microb Technol.

[B18] Salagnad C, Godde A, Ernst B, Kragl U (1997). Enzymatic large-scale production of 2-keto-3-deoxy-*D-glycero-D-galacto*-nonopyranulosonic acid in enzyme membrane reactors. Biotechnol Prog.

[B19] Wang TH, Lee WC (2006). Production of 2-keto-3-deoxy-*D-glycero-D-galacto*-nonopyranulosonic acid (KDN) using fusion protein of *N*-acetyl-*D*-neuraminic acid aldolase. Biochem Eng J.

[B20] Wang TH, Lee WC (2006). Expression and characterization of the *N*-acetyl-*D*-glucosamine 2-epimerase as a tagged protein for the conversion of *N*-acetyl-*D*-glucosamine to *N*-acetyl-*D*-mannosamine. J Chin Inst Chem Engrs.

[B21] Lee WC, Pan HH, Chen YY, Lee CH, Lin YS, Flynne WG, Nova (2008). Advances in enzyme immobilization through the use of recombinant DNA technology. Biotechnology and Bioengineering.

[B22] Kawakami B, Kudo T, Narahashi Y, Horikoshi K (1986). Genetic and molecular analyses of *Escherichia coli N*-acetylneuraminate lyase gene. J Bacteriol.

[B23] Uchida Y, Tsukada Y, Sugimori T (1984). Purification and properties of *N*-acetylneuraminate lyase from *Escherichia coli*. J Biochem (Tokyo).

[B24] Zimmermann V, Hennemann HG, Daussmann T, Kragl U (2007). Modelling the reaction course of N-acetylneuraminic acid synthesis from N-acetyl-D-glucosamine-new strategies for the optimisation of neuraminic acid synthesis. Appl Microbiol Biotechnol.

[B25] Dellaporta SL, Wood J, Hicks JB (1983). A plant DNA minipreparation: Version II. Plant Mol Biol Rep.

[B26] Takahashi S, Kumagai M, Shindo S, Saito K, Kawamura Y (2000). Renin inhibits N-acetyl-D-glucosamine 2-epimerase (renin-binding protein). J Biochem.

